# From Hemorrhagic Shock to Postoperative Complications: Anesthesia, Intensive Care, and Surgical Management of a Young Male With Pulmonary Aspergillosis

**DOI:** 10.7759/cureus.73017

**Published:** 2024-11-04

**Authors:** Lorena B Vargatu - Dinica, Mihai Sava, Alina S Bereanu, David L Achim, Stefan A Bancila, Corina R Seceleanu, Bogdan I Vintila

**Affiliations:** 1 Medical School, Faculty of Medicine, Lucian Blaga University, Sibiu, ROU; 2 Anesthesia and Critical Care, County Clinical Emergency Hospital/Faculty of Medicine, Lucian Blaga University, Sibiu, ROU; 3 Thoracic Surgery, County Clinical Emergency Hospital, Sibiu, ROU; 4 Anesthesia and Critical Care, County Clinical Emergency Hospital, Sibiu, ROU; 5 Anesthesia and Critical Care, County Clinical Emergency University/Faculty of Medicine, Lucian Blaga University, Sibiu, ROU

**Keywords:** bronchopleural fistula, hemoptysis, peri-operative medicine, pulmonary aspergillosis, thoracic anesthesia

## Abstract

Pulmonary aspergillosis is a life-threatening condition, especially for individuals with predisposing factors such as prior tuberculosis, smoking, and immune impairment. This case report describes the management of a 33-year-old male with a history of cured tuberculosis and active pulmonary aspergillosis who developed hemorrhagic shock following severe hemoptysis. Initial management included blood transfusion and the administration of tranexamic acid. Surgical prehabilitation was performed, and the patient underwent bilobectomy. Post-surgery, the patient developed respiratory failure due to a bronchopleural fistula, which was surgically repaired. The patient was managed in the intensive care unit (ICU) and subsequently discharged in good health. This case highlights the complexity of managing severe hemoptysis and its complications, and the importance of early multidisciplinary intervention and preoperative optimization.

## Introduction

Pulmonary aspergillosis encompasses a spectrum of fungal lung infections caused by *Aspergillus* species, with *Aspergillus fumigatus* being the most common strain affecting humans. The disease can manifest in various forms, from allergic reactions to more invasive forms leading to respiratory failure. This condition predominantly affects immunocompromised individuals, such as those with prolonged neutropenia, organ transplant recipients, patients on long-term corticosteroid therapy, previous cavitary lung disease, or patients with liver disease [1,2].

Pulmonary aspergillosis poses a significant challenge for diagnosis and treatment due to the limited accuracy of available tests, resulting in delays in proper management. Additionally, the condition is complicated by limited effective treatment options, the potential for significant drug interactions, adverse effects, superimposed bacterial infections, and the increasing issue of drug resistance [3-5].

The diagnosis of pulmonary aspergillosis is made based on the presenting symptoms (shortness of breath, cough, fever, chest pain, hemoptysis), relevant respiratory history, physical examination (respiratory sounds, signs of respiratory distress), initial blood tests (blood count, inflammatory markers, serological tests), microbiological studies, and imaging. The European Organization for Research and Treatment of Cancer and Mycoses Study Group Education and Research Consortium has established definitions that rely on host criteria (immunosuppressive conditions), clinical criteria (clinical and radiological signs as nodules, mass +/- halo sign), and mycological criteria (culture from bronchial aspirate or sputum, or positive galactomannan) [3,6,7]. 

Regarding the therapeutic approach, three antifungal drug classes are currently authorized to manage aspergillosis: polyenes, triazoles, and echinocandins. Ongoing research focuses on developing novel antifungal drugs to address the limited therapeutic options against aspergillosis-resistant cases. Addressing chronic pulmonary aspergillosis is complex and requires a multidisciplinary approach involving intensivists, pulmonologists, thoracic surgeons, radiologists, and infectious disease specialists [3,8,9].

This case report describes the clinical course of a young man who developed acute respiratory failure due to pulmonary aspergillosis and hemoptysis, complicated by a history of pulmonary tuberculosis and a weakened immune system. Despite initial treatment in the intensive care unit, the patient needed surgery to control the infection effectively. The particularity of the case involved managing severe hemoptysis without using bronchial artery embolization, emergency surgical repair of a bronchopleural fistula, and the use of prehabilitation to improve the patient's condition before surgery.

## Case presentation

We present the case of a 33-year-old Caucasian male who was transferred to our ICU from the Pneumonology Hospital due to hemorrhagic shock secondary to severe hemoptysis. The patient was initially admitted to the Pneumonology Hospital with complaints of a mild productive cough and moderate hemoptysis. His medical history was significant for aspergilloma localized in the right upper and middle lobes, a previous diagnosis of pulmonary tuberculosis, declared cured in 2017, as well as pulmonary fibrosis, bilateral pulmonary emphysema, and a history of smoking.

Upon admission, the patient presented with hemodynamic instability, necessitating the administration of vasoactive support with norepinephrine at a dose of 0.4 mcg/kg/min. Lung-protective ventilation was initiated via endotracheal intubation. The patient received two units of crossmatched blood, and a continuous infusion of tranexamic acid was initiated. Following evaluation by the thoracic surgery team, it was determined that surgical intervention would be deferred until stabilization and remission of the acute symptoms. The infectious diseases specialist recommended initiating antifungal therapy with voriconazole.

The thoracic computed tomography (CT) scan showed the presence of pulmonary emphysema, mainly affecting the upper regions of the lungs. Additionally, there were fibrotic lesions and cavities in the right upper lobe, along with areas of lung consolidation in the same region. The left upper lobe shows signs of bronchiectasis. The scan also indicateed micro-hemorrhages and a small pericardial effusion.

During the fiberoptic bronchoscopy performed through the orotracheal tube, viscous, purulent secretions that were more abundant on the right side were found in both lungs. Samples of the secretions were collected for microbiological analysis, and bronchoalveolar lavage was performed. There were no signs of active bleeding or procedural complications during the examination. After the procedure, sedation and neuromuscular blockade were discontinued, allowing for the safe extubation of the patient without complications.

Microbiological analysis of the collected pulmonary secretions confirmed the presence of *Enterobacter cloacae*. Based on this finding, the infectious disease specialist recommended initiating antibiotic therapy with moxifloxacin, linezolid, and amikacin.

The patient's ICU course was complicated by episodes of agitation, which were attributed to nicotine and alcohol withdrawal, as well as the underlying critical illness. Management of these episodes included the administration of benzodiazepines, continuous propofol infusion, haloperidol, and beta-blockers, alongside supportive measures such as frequent visits from the patient's family.

**Figure 1:**
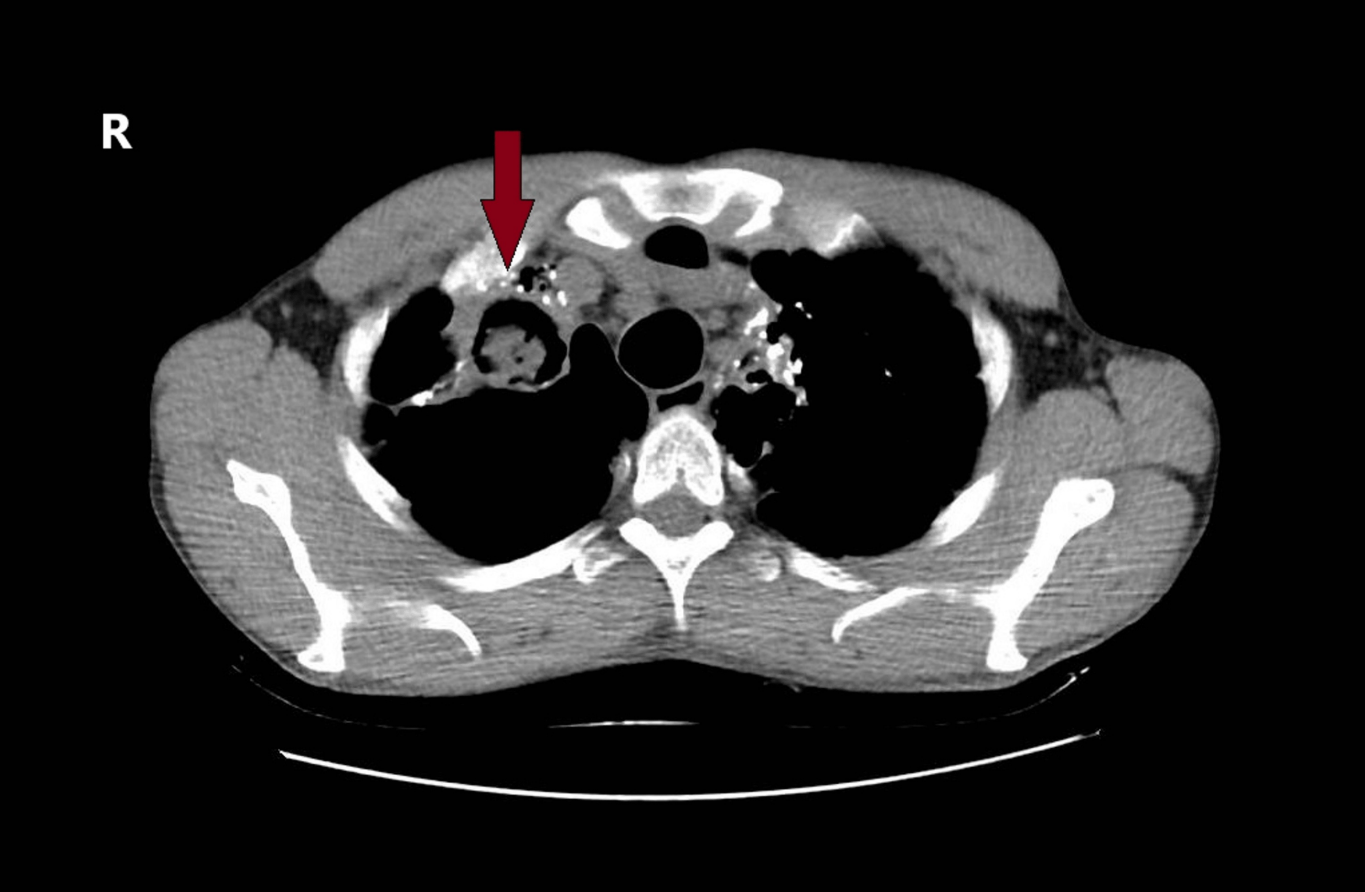
Transverse section of the preoperative thoracic computed tomography scan Right lung cavitary lesion (upper and middle lobes) with typical features of aspergilloma (air crescent sign - a crescent-shaped airspace separating the mass from the cavity wall - red arrow)

**Figure 2:**
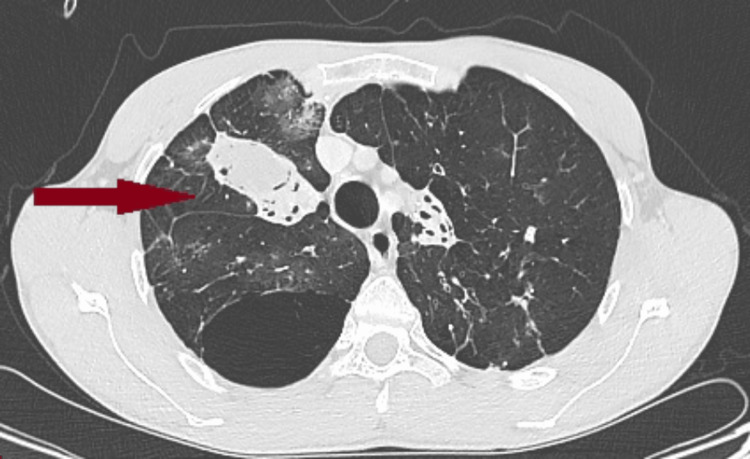
Transverse section of the preoperative thoracic CT scan showing a lesion in the right lung, involving both the upper and middle lobes (red arrow).

After an 11-day ICU stay marked by favorable clinical evolution, the patient was discharged in stable condition and returned one month later for a scheduled upper bilobectomy. The preoperative evaluation involved a multidisciplinary collaboration between anesthesia and intensive care, thoracic surgery, pulmonology, and cardiology departments. The echocardiographic assessment revealed mild mitral regurgitation (grade I), minor aortic insufficiency, and tricuspid regurgitation, with no evidence of pulmonary hypertension. The preoperative CT scan revealed typical features of aspergilloma (Figures 1, 2).

Two units of crossmatched packed red blood cells (PRBC) were reserved at the transfusion unit before surgery. The patient was brought to the operating room with stable vital signs. Anesthesia induction was performed using intravenous sufentanil (10 mcg), propofol (100 mg), and succinylcholine (100 mg), followed by the placement of a left-sided double-lumen endotracheal tube and a jugular central line. A right anterolateral thoracotomy was performed through the fourth intercostal space to access the pleural cavity and facilitate the superior bilobectomy. General anesthesia was maintained with intravenous sufentanil (total dose 120 mcg), rocuronium (total dose 130 mg), sevoflurane, and intermittent boluses of ephedrine as needed. The anesthetic course was uneventful, and following surgery, the patient was transferred to the ICU for postoperative monitoring and management. The patient was extubated four hours postoperatively.

**Figure 3:**
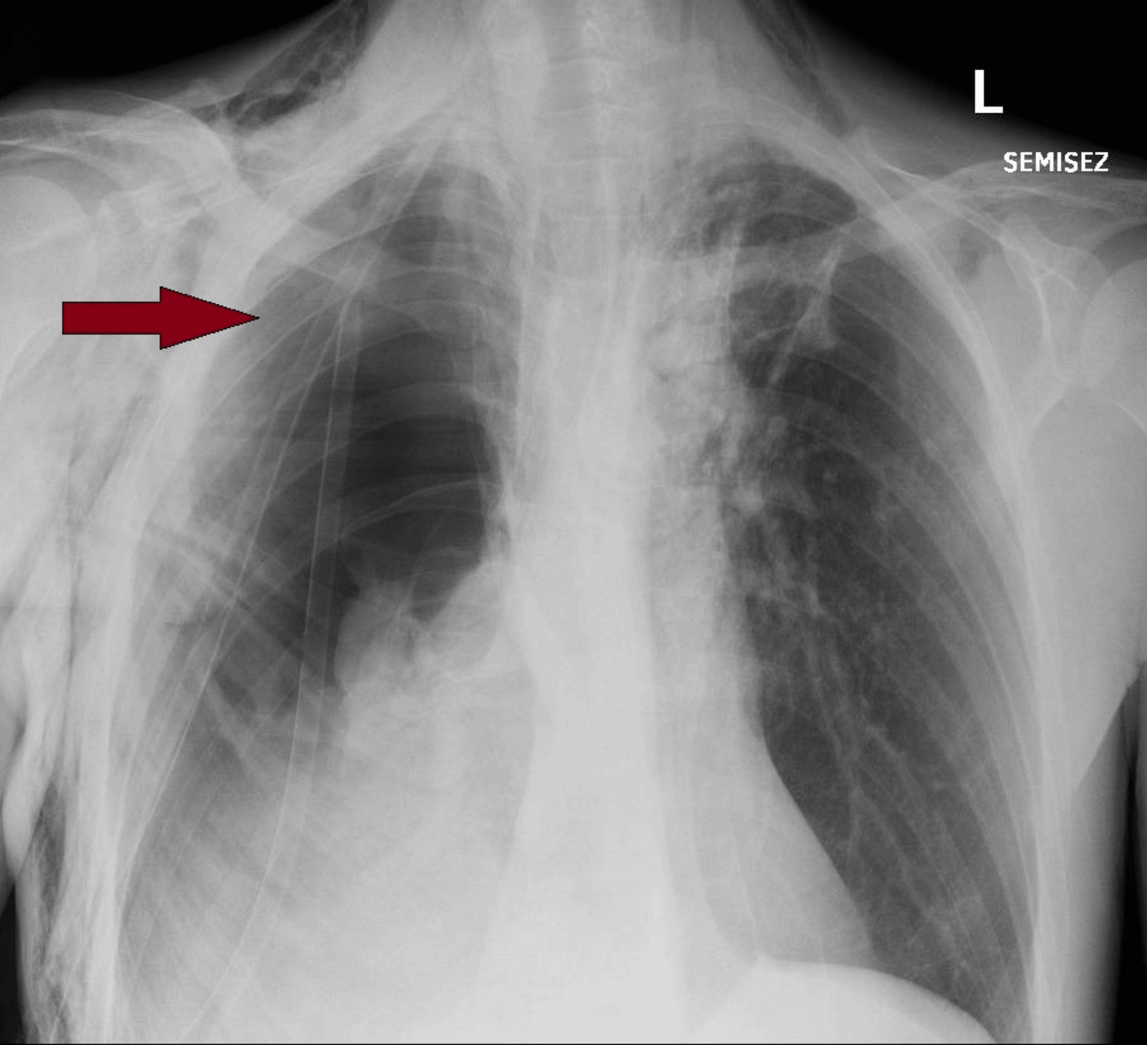
Anterior-posterior view of the chest X-ray on postoperative day 2 – Collapsed lung at the hilum with subcutaneous emphysema - red arrow.

On the second day after surgery, the patient developed respiratory failure, altered general status, and subcutaneous emphysema with significant air leakage from the chest drain. A bronchopleural fistula was suspected, and noninvasive mechanical ventilation was initiated to stabilize the patient while awaiting the chest X-ray. Although the patient's respiratory parameters showed improvement, the air leakage from the chest drain persisted. The chest X-ray showed a large right pneumothorax and a deterioration in the overall pulmonary radiographic findings (Figure 3). A bronchopleural fistula was suspected, and emergency reintervention was decided. Intraoperatively, a defect was identified at the bronchial stump of the right upper lobar bronchi, characterized by the absence of staples. A suture repair of the bronchial stump was performed and covered with pediculated intercostal muscle flap. The anesthetic course was uneventful, and the patient was transferred to the ICU postoperatively. Extubation of the trachea was safely achieved a few hours after surgery.

**Figure 4:**
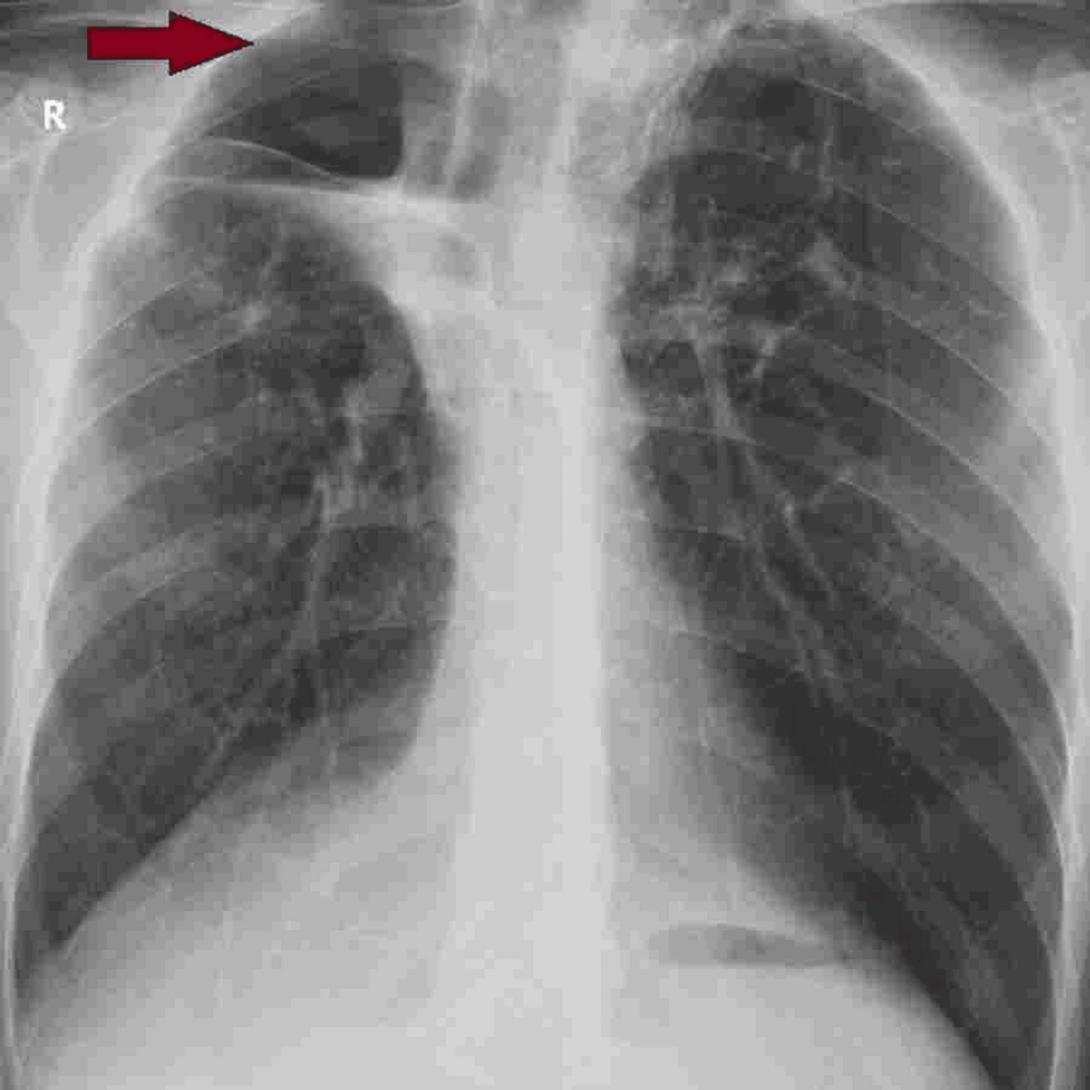
Posterior - anterior view of the chest X-ray one month postoperatively – Expanded lungs with a right apical cavity showing minimal fluid levels - red arrow.

During his stay, a severe vitamin D deficiency was also identified, for which vitamin D supplementation was initiated as part of the treatment regimen. After eight days of ICU care, the patient was discharged, and he consistently attended scheduled follow-up appointments at the thoracic surgery department. The patient remains in good health without any associated complications (Figure 4).

## Discussion

Nearly two million cases of pulmonary aspergillosis are diagnosed each year worldwide, with a high mortality rate within the first year. Over 50% of these patients experience significant hemoptysis. Bronchial artery embolization (BAE) is a safe and minimally invasive intervention for controlling hemoptysis, typically used when surgical management is delayed or unsuitable. However, the recurrence rate of hemoptysis after BAE is significant, approaching 50%. Tranexamic acid is effective in reducing bleeding in various clinical settings and is often used to manage hemoptysis, although evidence supporting its use in pulmonary aspergillosis is limited [10,11]. In this case, the patient's hemoptysis was effectively managed with blood transfusion and administration of tranexamic acid. Although BAE is the preferred intervention in managing significant hemoptysis, the cessation of hemorrhage and the procedure’s substantial recurrence rate led to a more conservative approach. Consequently, the patient was proposed for definitive surgical treatment following optimal preoperative preparation and prehabilitation.

The most common underlying conditions that predispose individuals to pulmonary aspergillosis include pulmonary tuberculosis, which is one of the most significant risk factors, as well as pulmonary fibrosis, emphysema, and chronic obstructive pulmonary disease, among others. Immune impairment is also a well-recognized risk factor for developing *Aspergillus* infections, as these fungi primarily affect individuals with compromised immune defenses [12,13]. Our patient is a young individual who was weakened due to a history of pulmonary tuberculosis, chronic smoking, and severe vitamin D deficiency. These factors together increase his susceptibility to both pulmonary aspergillosis and nosocomial infections.

Bronchopleural fistula (BPF) is a rare but severe complication following lobectomy, with an incidence reported in the literature ranging from 0.6% to 4%. Despite its low occurrence, BPF is associated with a high mortality rate, varying between 16% and 72%. Due to its complexity, early recognition and management of this complication presents significant challenges for clinicians and surgeons. Pulmonary infections (e.g., tuberculosis, aspergillosis), technical challenges during surgery, and factors that impair healing, such as chronic lung disease, malnutrition, immunosuppression, and smoking, increase the risk of bronchopleural fistula. In our case, the development of a bronchopleural fistula was likely due to a combination of these factors. [14-16]. Key components of treatment include initial chest tube drainage, administration of intravenous antibiotics to control infection, optimization of nutritional status, and definitive closure of the fistula through surgical or endoscopic intervention [14-16]. In our case, the patient developed a bronchopleural fistula two days following bilobectomy, which was complicated by the onset of respiratory failure, necessitating noninvasive ventilation, which allowed us to stabilize the patient effectively while imaging investigations were completed and preparations for surgery were initiated. Management involved an emergency surgical intervention, during which the fistula was promptly identified and sutured, accompanied by chest tube drainage and supportive care. The patient’s favorable outcome highlights the critical importance of rapid multidisciplinary intervention in addressing the risks and complications of BPF following lobectomy.

Advancements in technology have reduced the invasiveness of thoracic surgery, but patient comorbidities and frailty have increased. Risk factors for postoperative complications include poor functional reserve, nutritional deficiencies, sarcopenia, physical inactivity, smoking, and alcohol consumption [17-19]. Prehabilitation programs aim to optimize medical management, prescribe exercise regimens, correct nutritional deficiencies, and promote healthier behaviors to enhance surgical outcomes, and these programs are integral to enhanced recovery after surgery (ERAS) protocols [17-19]. The patient had modifiable risk factors, like smoking, vitamin D deficiency, and cachexia, which are linked to poor postoperative outcomes. These factors contribute to frailty and susceptibility to complications after thoracic surgery. Although the patient didn't undergo a formal prehabilitation program, elements of prehabilitation were addressed by optimizing his medical status before surgery, including nutritional supplementation, addressing vitamin D deficiency, and physical exercise, which contributed to his overall recovery.

The manuscript has several limitations: it's based on a single-patient case report, lacks long-term follow-up data, and doesn't provide detailed microbiological data on the *Aspergillus* infection. While bronchial artery embolization was considered, it was not performed as the hemoptysis was effectively managed conservatively, and given the location of the pulmonary lesion, surgery was indicated as a definitive treatment option, limiting comparative discussion on its efficacy versus surgical intervention for severe hemoptysis.

## Conclusions

Thoracic surgery, especially upper bilobectomy, presents significant challenges for anesthetic management due to the interaction of hemorrhagic risk with respiratory and hemodynamic factors. In our case, the patient's recent history of intensive care admission, immunosuppression exacerbated by severe vitamin D deficiency, and susceptibility to both community-acquired and nosocomial infections further complicated perioperative management. By considering these factors and adopting a multidisciplinary approach, successful management of the present case was achieved, minimizing complications and optimizing surgical outcomes.
